# Long-Term Effect of Aspartame on Male Reproductive System: Evidence for Testicular Histomorphometrics, Hsp70-2 Protein Expression and Biochemical Status

**DOI:** 10.22074/ijfs.2020.6065

**Published:** 2020-07-15

**Authors:** Hojat Anbara, Mohammad Taghi Sheibani, Mazdak Razi

**Affiliations:** 1 Department of Basic Sciences, Faculty of Veterinary Medicine, University of Tehran, Tehran, Iran; 2 Department of Comparative Histology and Embryology, Faculty of Veterinary Medicine, Urmia University, Urmia, Iran

**Keywords:** Aspartame, *Hsp70-2*, Mice, Testis

## Abstract

**Background:**

Aspartame is one of the most commonly consumed artificial sweeteners that is widely used in foodstuffs. There are many debatable reports about aspartame toxicity in different tissues; however, on the subject of its effects on the reproductive system, few literatures are available. The present study was carried out for evaluating effects of aspartame on the reproductive system in male mice.

**Materials and Methods:**

In this experimental study, a total of 36 adult male mice were randomly divided into four groups of nine animals each. Three groups received aspartame at doses of 40, 80 and 160 mg/kg (gavage) for 90 days; also, a control group was considered. Twenty-four hours after the last treatment, animals were sacrificed. Then, body and testis weights, sperm parameters, serum testosterone concentration, total antioxidant capacity, and malondialdehyde (MDA) levels, antioxidant enzymes [superoxide dismutase (SOD), catalase (CAT) and glutathione peroxidase (GSH-Px)] activities in blood, histomorphometrical indices and histochemical changes in testis were evaluated; also, mRNA and immunohistochemical expression of Hsp70-2 was measured in testis tissue.

**Results:**

The results revealed remarkable differences in sperm parameters, testosterone and oxidative stress biomarkers levels, and histomorphometrical indices, between the control and treatment groups. Also, in 80 and 160 mg/kg aspartametreated groups, expression of Hsp70-2 was decreased. Besides, in the aspartame receiving groups, some histochemical changes in testicular tissue were observed.

**Conclusion:**

The findings of the present study elucidated that long-term consumption of aspartame resulted in reproductive damages in male mice through induction of oxidative stress.

## Introduction

Recently, food consumers are increasingly concerned about the quality and safety of many foodstuffs produced by industrialized countries; in particular, the usage of artificial sweeteners, flavorings, dyes, preservatives and food supplements has raised concerns. Many non-nutrient sweeteners have been used in foods and beverages to help people enjoy a sweet taste without raising body calories. One of these sweeteners is aspartame (1). Aspartame (Laspartyl-L-phenylalanine methyl ester) is a synthetic nonnutritional sweetener that was firstly discovered in 1965 and approved in 1981 for use in the United States (2). This sweetener is a dipeptide derived from the combination of two non-aromatic amino acids namely, aspartic acids and phenylalanine. Sweetening power of aspartame is 160 to 180 times more than that of sucrose, it has the same number of calories as sugar, and it does not smell and lacks metallic taste (3).

After oral intake, aspartame is hydrolyzed in the gastrointestinal tract by esterases and peptidases into amino acids (aspartic acid and phenylalanine) and methanol. Also, it is possible that aspartame is absorbed by the mucosal cells of the intestines and metabolized before hydrolysis (3).

Methanol is not metabolized in the enterocytes; it immediately enters the portal circulation and is then oxidized in the liver into formaldehyde (4). Metabolism of methanol into formaldehyde and formic acid is associated with formation of superoxide anion and hydrogen peroxide (5). Development of oxidative stress through methanol oxidation results in structural and functional impairments of proteins responsible for regulating and maintaining the lower temperature of the testes (7). In fact, the testicular temperature must be 2-4°C lower than the rest of the body for maintaining optimal sperm quality. Even a slight increase in temperature could lead to rapid disrupts in spermatogenesis due to inducing protein denaturation (8). Hsp70 proteins besides increasing the RNA-binding protein stability in haploid cells, are able to take part in recovering DNA and RNA damages through improvement of DNA integrity. During early meiosis and/or mitosis, Hsp70-2, which is considered the main expressed chaperone, has capability to induce folding/refolding in proteins during different phases of cell cycles. Also, there is evidence about more than 20 chaperone families, which are influenced by some biochemical stressors, including oxidative and nitrosative stresses, and become well up regulated (8).

Considering the fact that the majority of these chaperone families are cell stress responders or heat shock proteins (HSPs), chaperones have important roles in raising the cellular resistance against environmental stressors, although the HSPs are known to be involved in regulating spermatogenesis (8, 9). Researchers have reported that long-term consumption of aspartame up-regulates the expression of Hsp70 in the brain, heart, liver and immune organs (6, 10-12). Beside having cytoprotective effects, Hsp70 has a role in regulating spermatogenesis (8, 9). It was indicated that long-term consumption of aspartame leads to reproductive toxicity in male rats (13). In the present study, we investigated Hsp70-2 expression following long-term consumption of aspartame in male mice. Also, in order to confirm induction of oxidative stress and reproductive toxicity by aspartame, levels of oxidative stress biomarkers were measured in blood. Moreover, concentration of testosterone in serum was measured, and histochemical and histopathological evaluations in testis tissue were performed in order to confirm reproductive toxicity of aspartame. In the literature, there is no study done in the male genital system that investigated Hsp70, or performed histomorphometerical assessment in this context, which implies the novelty of the present experiment.

## Materials and Methods

### Chemicals

Aspartame was purchased from Sigma-Aldrich (St Louis, MO, USA, CAS No. 22839-47-0). Acridine orange was purchased from sigma chemical Co. (St. Louis, MO, USA). All other chemicals used were commercial products of analytical grade. The rabbit anti-mouse primary antibodies for *Hsp70-2* (Cat NO. SKU: 407), were obtained from Biocare (Biocare, California, USA).

### Animals

All experimental protocols were conducted on the basis of the proofed principles for laboratory animal care (7506025.6.24), approved by the Ethical Committee of the University of Tehran. For this study, a total number of 36 NMRI mature male mice (8-10 weeks of age), weighing 25-35 g were used. The animals were provided from the Laboratory Animal Sciences Center, Pasteur Institute of Iran, Karaj, Iran. Before initiation of the treatment period, the mice were maintained for two weeks in order to acclimatize. The mice were housed in special cages under well-ventilated conditions at normal temperature (22 ± 5°C) with 12:12-hour light-dark cycles and fed standard pellet diet (Tehran pellet, Iran).

### Chemical administration and grouping

In this experimental study, The European Food Safety Authority has confirmed acceptable daily intake (ADI) for 40 mg/kg bodyweight/day of aspartame. This ADI was approved by the food and drug administration (FDA) for the European countries (EFSA Journal 2013). After labeling the mice, they were randomly divided into four groups of nine mice. The treatment groups received aspartame for 90 days by gavage as follows:

1. The first group (control): The animals of this group received normal saline at the dosage of 0.5 ml.

2. The second group was called low dose aspartame and it received 40 mg/kg bodyweight/day of aspartame.

3. The third group was called medium dose aspartame and it received 80 mg/kg bodyweight/day of aspartame.

4. The forth group was called high dose aspartame and it received 160 mg/kg bodyweight/day of aspartame.

Thereafter, the animals were kept under standard conditions and monitored for 90 days. On the basis of the fact that the duration of the chronic dose of aspartame is ninety days to have probable pathogenicity, this period was chosen for this experiment. The dosages and duration of the treatment in the present study were chosen on the basis of earlier studies (13, 14) (Fig .S1, See Supplementary Online Information at www.ijfs.ir).

### Serum and tissue samples preparation

Following the 90-day period, all animals were anesthetized using a mixture of ketamine and xylazine cocktail (0.10 ml xylazine and 1 ml ketamine and 8.90 ml distilled water), with the dose of 0.1 ml/10 g BW (15). In order to obtain serum, 15 minutes after anesthesia induction, the blood samples were centrifuged at 3000 g for 10 minutes at room temperature (RT) and stored at -70°C for further analyses. The testicular specimens were removed and rinsed with chilled normal saline. One of the testes from each individual mouse was snap frozen in liquid nitrogen and then kept in -70°C until further biochemical analyses and the other testes were fixed in Bouin’s solution for histological examinations.

### Histomorphometrical and histochemical assay

The testes were quickly dissected out, cleared of adhered connective tissue and weighed on a digital scale (with a minimum accuracy of 0.001 g). For Histomorphometrical study, Dino-Lite digital lens and Dino Capture 2 Software were used. Furthermore, histometrical structures of the testes, including testicular capsule thickness, germinal epithelium height and diameter of seminiferous tubules, as well as the number of Sertoli and Leydig cells were evaluated. In order to classify spermatogenesis, Johnsen’s criteria were used. This classification is based on graded scoring between 1-10 for each tubule cross-section, according to presence or absence of main cell types organized in the order of maturity:10, complete spermatogenesis exists and tubules are normal in arrangement; 9, there are many spermatozoa with disorganization in germinal epithelium; 8, only a few spermatozoa are observed; 7, lacking spermatozoa while many spermatids exist; 6, only a few spermatids are present; 5, absence of spermatozoa and spermatids but existence of many spermatocytes; 4, only a few spermatocytes exist; 3, only spermatogonia are observed; 2, presence of only Sertoli cells and the absence of germ cells, and 1, no germ cells or Sertoli cells are present. Tubule cross-sections with scores of 9 and 10 were considered mature tubules (15).

Paraffin blocks were sectioned at 5-6 μm and stained with Hematoxylin and Eosin (H&E), Periodic acid-Schiff (PAS) and Masson's trichrome. Masson’s trichrome staining was used to show the amount of collagen fibers and fibrosis in testicular tissue. In order to analyze carbohydrate ratio in testicular germinal epithelium, PAS was conducted on specimens. Also, for the purpose of histochemical evaluations, frozen sectioning method was carried out. The samples were embedded using optimal cutting temperature compound (OCT gel) and sections of testicular tissues were prepared at 15-20 μm levels at -40°C using cryostat (SLEE, Germany). Also, the Sudan black B (SB) staining was performed to evaluate the rate of lipid foci supplement in treatment and control animals and identify the Leydig cells cytoplasmic bio-steroid supplement. The alkaline phosphatase staining (ALP) was conducted to demonstrate the ratio of this enzyme as a biomarker for inflammation. The photomicrographs were taken by a SONY on-board camera (Zeiss, Cyber-Shot, Japan).

### Sperm preparation and DNA damage assessment

Epididymides were carefully refined from their surrounding tissues under 10X magnification provided by a Stereo Zoom Microscope (TL2, Olympus Co., Tokyo). The caudate part of the epididymis was trimmed and minced in 5 ml TCM199 medium for 30 minutes, with 5% CO_2_, at 36.5°C in a CO_2_- equipped incubator (LEEC Co., England). After centrifugation, the sperm pellet was re-suspended in 0.5 ml of TCM199 medium. A small aliquot (20 μl) of sperm suspension was glass-smeared. The slides were air-dried and then fixed overnight in Carnoy’s solution (methanol/acetic acid, 3:1). Next, they were stained for 5 minutes with a freshly-prepared acridine orange stain (AO). After washing and drying, the slides were examined using a fluorescent microscope (Leitz, Germany; excitation of 450-490 nm). On each slide, an average of 200 sperms were evaluated and two types of staining patterns were identified including yellow (single-stranded DNA) sperms and green (double-stranded DNA) (16).

The percentage of spermatozoa with single-stranded DNA was calculated from the ratio of spermatozoa with red, orange, or yellow fluorescence, to the total spermatozoa counted per sample.

### Sperm count, motility and viability

Sperm count was assessed by a standard hemocytometer method (15). The motility of the sperm was evaluated according to the WHO (WHO, 2010) standard method for manual examination of sperm motility (17). Accordingly, the sperm samples were diluted 1:8 in TCM199 before the assessment. A 20-μl sample of the sperm was placed on a sperm test area and evaluated under 1,000X magnifications. Only the motile sperms with forward progression were counted within 10 boxes and recorded. Finally, motility was calculated based on the following equation: Motility (%) = [motile sperm/(motile+non-motile sperm)]×100.

The Eosin-nigrosin staining method was performed to assess the sperm viability. For this purpose, 50 μl of sperm was mixed with 20 μl of eosin in a sterile test tube. After 5 seconds, 50 μl of nigrosin was added and mixed thoroughly. Then, the mixture of the stained sperm was smeared on the slide and examined under a light microscope (1,000X magnification, Olympus, Germany). The colorless sperms were considered live and the yellow to pink stained sperms were marked as dead. The sperm count was performed according to the standard hemocytometric method previously described by Pant and Srivastava (16). The sperm viability and motility are reported in percentage and compared between groups.

### Assessment of serum levels of testosterone

Following 90 days, blood samples were obtained directly from the heart under light anesthesia (induced using diethyl ether). After 15 minutes, the samples were centrifuged at 3000×g for 10 minutes at RT to obtain serum. Serum concentration of testosterone was measured by enzyme-linked immunosorbent assay (ELISA) as described by the manufacturer (Demeditec Diagnostics GmbH, Germany). In brief, 100 μl of serum sample and control (from the kit) were dispensed into the ELISA wells, and 100 μl of Enzyme conjugate was added into the wells and thereafter, incubated 60 minutes at RT. Next, the content of the wells was discarded and rinsed 4 times with diluted Wash Solution (300 μl per well), and 200 μl of Substrate Solution was added to each ELISA well. The samples were thereafter incubated in the dark for 30 minutes. Finally, 50 μl of Stop Solution was added to each well and the absorbance of each sample was determined at 450 nm.

### Assessment of oxidative stress biomarkers

Some important detectable oxidative stress biomarkers, including total antioxidant capacity (TAC), and activities of antioxidant enzymes catalase (CAT), superoxide dismutase (SOD), glutathione peroxidase (GSH-Px), and malondialdehyde (MDA) and nitric oxide (NO) content were measured in the blood samples as described previously (15, 18).

Determination of GSH-Px activity was performed by GSH-Px detection kit (Ransel, RanDox Co, UK) based on manufacturer’s instructions. One unit of GSH-Px was defined as μM of oxidized NADPH per minute mg-1 of protein. A decrease in absorbance was recorded by spectrophotometry against blank, at 340 nm.

The SOD activity was evaluated at 505 nm using a standard curve. The SOD activity was determined by the SOD detection kit (RanSod, RanDox Co, UK) based on the manufacturer’s instructions. Serum NO level was measured according to the Griess reaction (17) and expressed as μM/l. CAT activity, on the basis of a previously described method, was evaluated. Here, the blood samples were homogenized in Triton X-100 1% (Merck, Germany) and then diluted using phosphate buffer (pH=7.0). For initiation of the reaction, hydrogen peroxide was added to the mixture and the level of enzyme activity on the basis of the competency of the CAT in decompensation of hydrogen peroxide, was determined. This was gained through scanning the decrease in absorbance at 240 nm against a blank containing phosphate buffer instead of the substrate. The value of log A1/ A2 of a measured interval was used for unit definition due to the initial reaction of the enzyme, where the value of A1 refers to the absorbance at 240, at time 0 seconds and A2 is the absorbance at 240, at second 15. These enzyme activities were expressed as U g-1 Hb in blood. Then the measurement of the protein level in supernatant took place using the colorimetric method of Lowry with bovine serum albumin (BSA) as standard (15).

The MDA level as an indicator of lipid peroxidation in serum was determined according to the procedure described by Buege and Aust. Here, 100 μl of serum specimens using a glass homogenizer was homogenized in 0.15 M/l KCl at a ratio of 1 to 9 ml. One volume of homogenate was blended thoroughly with two volumes of a stock solution of 15% w/v trichloroacetic acid, 0.375% w/v thiobarbituric acid, and 0.25 M/l hydrochloric acid. After heating and cooling cycles, the solution was clarified by centrifugation at 1000 ×g for 10 minutes. The absorbance of the clear solution was read at 535 nm and MDA content was figured out using 1.56 ×10^5^ M-1 cm-1 as molar absorbance coefficient. MDA levels are presented as mM per ml protein (15).

Evaluation of TAC was carried out on the basis of the manual of the kit (TAS test kit, Randox Laboratories Ltd, GB).

### Immunohistochemical analysis for Hsp70-2

Immunohistochemical staining was done in order to analyze Hsp70-2 positive cells distribution. For this, before beginning the staining process, 5-μm tissue sections were heated at 60°C for approximately 25 minutes in a hot-air oven (Venticell, MMM, Einrichtungen, Germany). After deparaffinization in two changes of xylene, the sections were rehydrated using an alcohol gradient (96, 90, 80, 70, and 50%). The antigen retrieval process was performed in 10 mM sodium citrate buffer (pH=7.2). Immunohistochemical staining was conducted according to the manufacturer’s protocol (Biocare, USA). In brief, endogenous peroxidase was blocked in a peroxidase blocking solution (0.03% hydrogen peroxide containing sodium azide) for 5 minutes. Washing the sections was done with phosphate-buffered saline (PBS, DNAbiotech, Iran, pH=7) and subsequently incubation was performed with Hsp70-2 (1:600) biotinylated primary antibodies (Biocare, USA) at 4°C in humidified chamber overnight. After rinsing with PBS, the sections were incubated with streptavidin–HRP (streptavidin conjugated to horseradish PBS containing an anti-microbial agent) for 20 minutes. Followed by rinsing in washing buffer and adding a 3,3' Diaminobenzidine (DAB) chromogen, they were incubated for 10 minutes and counter stained with hematoxylin for 10 seconds. Then, the sections were dipped in ammonia (0.037 Ml), rinsed with distilled water, and cover slipped. Positive immunohistochemical staining could be observed as brown stains under a light microscope (8).

### RNA isolation, cDNA synthesis and reverse transcription -polymerase chain reaction

For RNA extraction, the collected testicles and those previously stored at -70°C, were used; RNA extraction was performed on the basis of the standard TRIzol method (8). For this, 20-30 mg of testicular tissue from an individual animal of each group was homogenized in 1 ml of TRIZOL. Then, in order to avoid genomic DNA contamination, the colorless aqueous phase was collected carefully. The quantitative assessment of RNA was performed using a nanodrop spectrophotometer (260 nm and A260/280=1.8-2.0), followed by storage of the samples at -70°C. For reverse transcriptionpolymerase chain reaction (RT-PCR), the cDNA was synthesized in a 20-μl reaction mixture containing 1 μl of oligo (dT) primer, 1 μl of RNAse inhibitor, 4 μl of 5X reaction buffer, 1 μg of RNA, 1 μl of M-MuLV reverse transcriptase and 2 μl of a 10 mM dNTP mix, on the basis of the manufacturer’s protocol (Fermentas, GmbH, Germany). The cycling protocol for 20 μl reaction mix was 5 minutes at 65°C, followed by 60 minutes at 42°C, and 5 minutes at 70°C to finalize the reaction. For evaluating the PCR reaction, a total volume of 27 μl containing primers pair's sequences (each 1 μl), 13 μl of PCR master mix and cDNA as a template (1.5 μl) and 10.5 μl of nuclease free water, were used. The following PCR conditions were considered; general denaturation at 95°C for 3 minutes for 1 cycle, followed by 35 cycles of 95°C for 20 seconds; annealing temperature (62°C for *Hsp70-2*, and 58°C for *GAPDH*) for 60 which participate in antioxidant defense system (6). Hsp70 functions as a cell supportive factor against many stresses that induce the production of reactive oxygen species(ROS), which in turn affect cellular molecules including DNA, proteins and lipids. Also, Hsp70 protein is known to be seconds; elongation: 72°C for 1 minute and 72°C for 5 minutes. Final PCR products were analyzed on 1.5% agarose gel electrophoresis and densitometric analysis of the bands were done by using PCR Gel analyzing software (ATP, Iran). The control was set at 100% and experimental samples were compared to the control. Specific primers were designed and manufactured by Sinaclon (Sinaclon Co., Iran). The primers pair's sequences and product size for primers used in RT-PCR are presented in Table 1.

**Table 1:** Nucleotide sequences and product size of primers used in reverse transcription-polymerase chain reaction

Target gene	Primer sequence (5'-3')	Product size (bp)

*Hsp70*	F: CAGCGAGGCTGACAAGAAGAA	340
	R: GGAGATGACCTCCTGGCACT	
*GAPDH*	F: TGAAGCAGGCATCTGAGGG	320
	R: CGAAGGTGGAAGAGTGGGAG	


### Statistical analysis

The data was analyzed using SPSS program version 19.0 (SPSS Inc, Chicago, IL, USA). All results are presented as mean ± SD. Differences between quantitative histological and biochemical data were analyzed by oneway ANOVA, followed by Tukey test, using Graph Pad Prism, 4.00. The P<0.05 were considered statistically significant.

## Results

### Histomorphometrical parameters

The results of histomorphometric studies showed that the thickness of testicular capsule in the high-dose group of aspartame, had a significant increase compared to the control group, whereas, the number of Sertoli and Leydig cells showed a significant decrease (P<0.05) in this group. Also, in medium- and high-dose aspartame-treated groups, a significant decrease (P<0.05) was observed in the diameter of the seminiferous tubules, the height of the germinal epithelium and the Johnsen’s score (Table 2).

### Histological and histochemical findings

**Fig.1:**
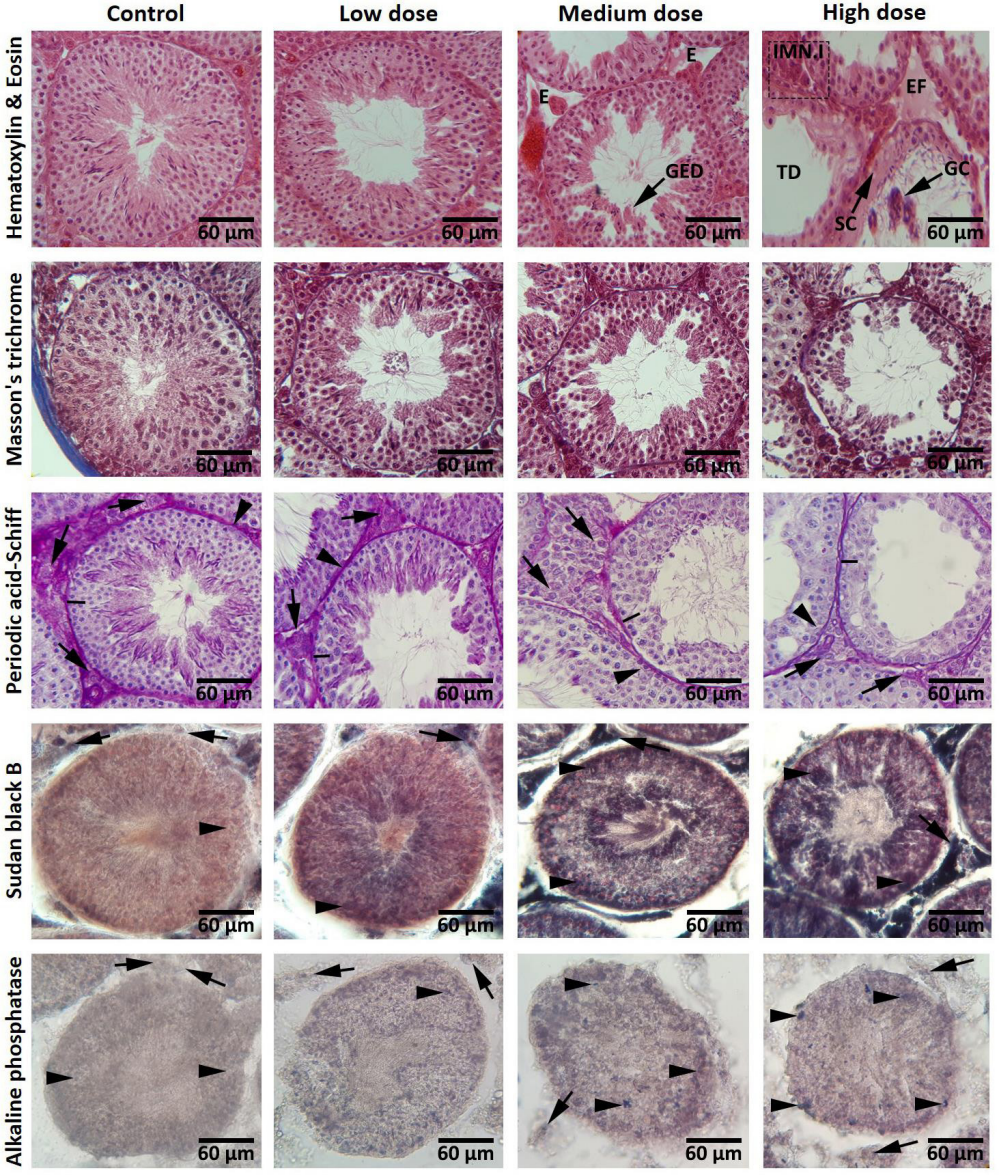
Cross sections from testes: Hematoxylin & Eosin staining; intact spermatogenesis is seen in the control group. Cross sections from medium- and high-dose groups present reduced epithelial height as well as germinal epithelium dissociation (GED), edema (E) and oedematous fluid accumulation (EF) of interstitial connective tissue, immune cells infiltration (IMN.I), atrophic and depletion seminiferous tubules (TD), giant cell (GC), detachment of Sertoli cell (SC) and spermatogenesis. Masson’s trichrome staining; there was no difference in the amount of collagen fibers between the control group and the aspartame-treated groups. Periodic acid-Schiff staining; Control group with the Leydig cells (arrows), Sertoli cells (head arrows) and the first three cell layers (lines) with normal Periodic acid-Schiff (PAS) reaction. Low-dose group with light germinal cell dissociation and moderated PAS reaction are present in seminiferous tubules. Medium- and high-dose groups with negative PAS reaction in Leydig cells (arrows), Sertoli cells (head arrows) and the first three cell layers (lines) with faint PAS-stained cytoplasm. Sudan black B staining; Frozen sections from testes. Control group with spermatogenesis series cell lineage with negative Sudan black B-stained cytoplasms (arrows) and Leydig cells area (head arrows) are appeared with dense reaction sites. Comparing aspartame-treated groups with the control group indicates that in low-dose group, spermatogenesis series cells are presented with faint lipid stained cytoplasms (arrows) and Leydig cells area (head arrows) stained densely, while the medium- and high-dose groups are manifested with darkly stained spermatogenesis series cells (arrows) and Leydig cells area (head arrows). Alkaline phosphates staining; Frozen sections from testes. All germinal epithelium cells (head arrows) and Leydig cells area (arrows) in the control group are presented with the negative alkaline phosphatase (ALP) reaction. Comparing the aspartame-received groups reveals that there are numbers of cells in the germinal epithelium (head arrows) and Leydig cells (arrows) with ALP-stained cytoplasms (scale bar: 60 μm).

Our histological observations revealed that aspartame, in a dose-dependent manner, could increase disarrangement and produce severe edema in connective tissue. An increase in germinal epithelium dissociation (GED) and tubular depletion in medium- and high-dose aspartametreated groups, was observed. Especially in the highdose group, aspartame could induce drastic morphologic changes in the testes. There were some atrophied seminiferous tubules indicating severe reduction in the number of germ cells and intensive immune cells infiltration, edematous fluid accumulation and intertubular space widening in interstitial connective tissue. Moreover, Sertoli cells lost their junction with germ cells and looked amorphous with irregular and smaller nuclei (Fig .1).

Also, concerning the histochemical features observed following Masson’s trichrome staining, it was found that the groups do not differ in the density of collagen fibers. Histochemical analyses of the PAS-stained specimens elucidated that the cells in three first layers of spermatogenesis cell series, Sertoli and Leydig cells faintly reacted with PAS in medium- and high-dose aspartame-treated groups and the carbohydrate ratio was severely decreased in their cytoplasm. In Sudan black B staining, in seminiferous tubules, brown to black particles which contain lipid were clearly seen inside the cytoplasm of the cells close to the lumina of seminiferous tubules and Leydig cells. No cytoplasmic lipids in Leydig cells and spermatogenesis series cells in the control group, were observed. Animals in the aspartame receiving groups showed high lipid-stained sites in the cytoplasm of the Leydig cells and spermatogenesis series cells. In testicular tissue section, alkaline phosphates staining indicated the highest rate of small brown particles in the cytoplasm of Leydig cells and spermatogenesis cells in the high-dose group, compared to the other groups. In addition, it should be noted that the level of alkaline phosphatase reaction in the groups treated with aspartame was dose-dependently increased (Fig .1).

**Table 2:** Comparison of sperm parameters (± SD) between the experimental groups after frozen-thawed and treatment with 10 μg/ml Calligonum (CGM) extract and LIPUS (pulsed mode and continues wave)


Parameters	Control	Low dose	Medium dose	High dose

TBW (g)	36.12 ± 2.82^a^	36.31 ± 3.06^a^	36.38 ± 3.67^a^	37.31 ± 3.01^a^
TW (g)	0.12 ± 0.011^a^	0.12 ± 0.009^a^	0.12 ± 0.008^a^	0.10 ± 0.010^b^
BWA (g)	4.62 ± 0.67^a^	5.24 ± 1.56^ab^	5.58 ± 2.53^ab^	6.97 ± 1.15^b^
Testosterone (ng/ml)	6.88 ± 0.32^a^	6.44 ± 0.30^a^	6.22 ± 0.53^a^	5.10 ± 0.57^b^
STsD (μm)	194.38 ± 4.33^a^	187.48 ± 5.56^a^	173.32 ± 5.78^b^	161.96 ± 5.45^c^
GEH (μm)	58.97 ± 3.48^a^	57.36 ± 2.36^a^	50.02 ± 1.79^b^	43.69 ± 3.28^c^
TCT (μm)	13.47 ± 1.27^a^	14.07 ± 2.09^a^	16.41 ± 1.93^a^	20.70 ± 2.58^b^
LCs (No/mm^2^)	37.35 ± 2.79^a^	36.82 ± 2.11^a^	33.57 ± 2.30^a^	28.72 ± 2.97^b^
SCs (No/one tubule)	22.76 ± 1.37^a^	22.79 ± 1.64^a^	20.72 ± 1.83^a^	16.68 ± 1.55^b^
Johnsen’s score	9.42 ± 0.26^a^	9.35 ± 0.30^a^	8.64 ± 0.39^b^	7.52 ± 0.47^c^
Sperm count (×10^6^)	34.66 ± 1.65^a^	31.55 ± 1.42^b^	27.44 ± 1.81^c^	19.22 ± 1.48^b^
Sperm motility (%)	85.06 ± 2.32^a^	81.95 ± 3.32^a^	74.34 ± 1.25^b^	62.40 ± 2.98^c^
Sperm viability (%)	89.22 ± 1.56^a^	86.66 ± 2.73^a^	79.33 ± 1.80^b^	72.55 ± 2.12^c^
DNA damage sperms (%)	5.11 ± 1.36^a^	7.55 ± 1.94^a^	11.22 ± 2.16^b^	19.33 ± 2.12^c^
Abnormal sperms (%)	10.33 ± 0.86^a^	11.66 ± 1.22^a^	15.44 ± 1.87^b^	19.88 ± 1.69^c^


All data are presented as mean ± SD. Low dose; 40 mg/kg aspartame-treated, Medium dose; 80 mg/kg aspartame-treated, High dose; 160 mg/kg aspartame-treated. TBW; Total body weight, TW; Testicular weight, BWA; Body weight alternations, STsD; Seminiferous tubules diameter, GEH; Germinal epithelium height, TCT; Testicular capsule thickness, LCs; Leydig cells, and SCs; Sertoli cells. Different superscripts in the same row show significant differences between groups (P<0.05).

### Sperm characteristics

**Fig.2:**
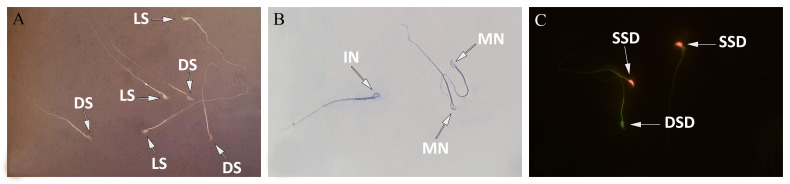
Photomicrographs of mice epididymal spermatozoa. **A.** Eosin-nigrosin staining, **B.** Aniline-blue staining, and **C.** Acridine-orange staining, (1,000X). DS; Dead sperms, LS; Live sperms, MN; Mature nucleus, IN; Immature nucleus, SSD; Single-strand DNA, and DSD; Double-strand DNA.

Observations showed that aspartame in a dose-dependent manner, significantly (P<0.05) reduced the sperm count. Survival rate and sperm motility in medium- and high-doses aspartame-treated groups were significantly decreased (P<0.05) compared to the control group. Also, the average percentage of abnormal sperms as well as the percentage of sperms with damaged DNA, in medium- and high-dose aspartame-treated groups was significantly increased (P<0.05, Fig .2, Table 2).

### Effect of aspartame on oxidative stress parameters

**Fig.3:**
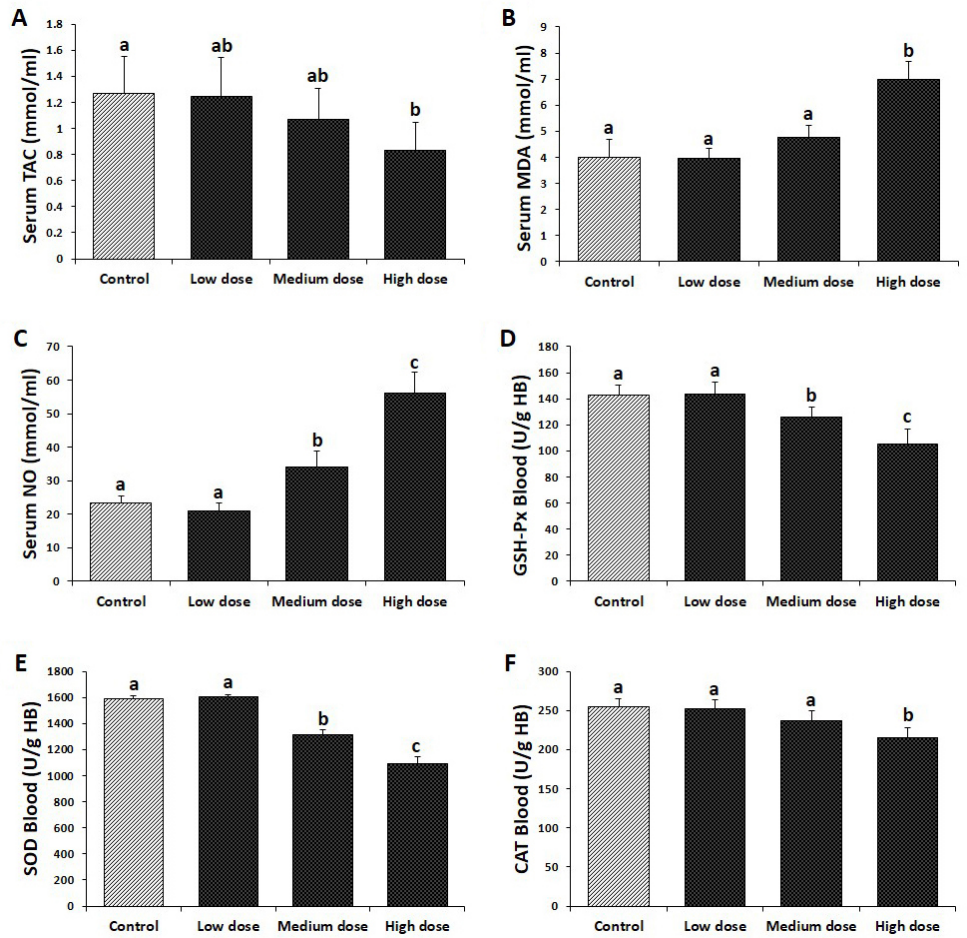
Effect of aspartame on antioxidant status. **A.** Serum total antioxidant capacity (TAC), **B.** Serum malondialdehyde (MDA) level, **C.** Serum nitric oxide (NO) level, **D.** blood glutathione peroxidase (GSH-Px) activity, **E.** Blood superoxide dismutase (SOD) activity, and **F.** Blood catalase (CAT) activity in different groups. All data are presented as mean ± SD. The different superscripts are representative of significant differences (P<0.05) between groups. Low dose; 40 mg/kg aspartame-treated, Medium dose; 80 mg/kg aspartame-treated, and High dose; 160 mg/kg aspartame-treated.

Aspartame effects on various parameters of oxidative stress biomarkers in serum and blood samples are shown in Figure 3. As can be seen, aspartame administration resulted in a significant increase (P<0.05) in MDA levels in the high-dose group as well as NO in medium- and high-dose aspartame-treated groups compared to the control group. Also, our observations showed that aspartame could induce a significant decrease (P<0.05) in TAC and CAT activity in the highdose group and consequently led to a significant decrease (P<0.05) in the level of GSH-Px and SOD in both medium- and high-dose aspartame-treated groups compared to the control group.

### Aspartame diminished Hsp70-2 expression

**Fig.4:**
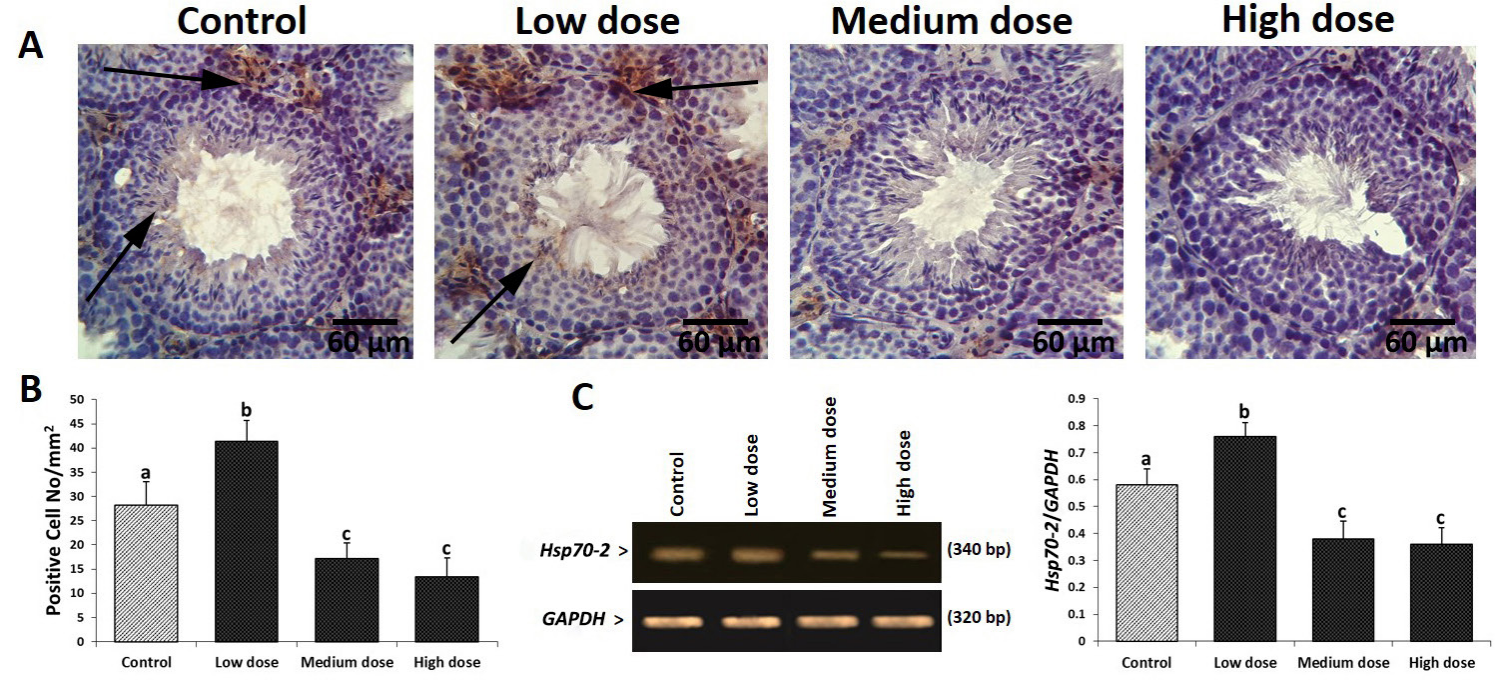
Effect of aspartame on Hsp70-2 protein expression in different groups. **A.** Immunohistochemical staining for Hsp70-2; see arrows indicating positive reaction for Hsp70-2 in two cell lines of genital cells in the control group, which is elevated in all cellular layers of low-dose group and significantly decreased in medium- and high-dose groups, respectively. **B.** See cell count results for Hsp70-2 (+) cells/1 mm2 of tissue in different groups. **C.** The mRNA levels of Hsp70-2 and GAPDH were evaluated by using semi-quantitative reverse transcription-polymerase chain reaction (RT-PCR). The density of Hsp70-2 mRNA levels in testicular tissue was measured by densitometry and normalized to GAPDH mRNA expression level. Data are presented as mean ± SD. The different superscripts are representative of significant differences (P<0.05) between groups.

The mRNA and protein levels of Hsp70-2 were analyzed. In order to clarify Hsp70-2 expression in different cellular layers of germinal epithelium, immunohistochemical analyses were done. Our finding revealed that, biosynthesis of Hsp70-2 increased in low-dose aspartame-treated group (especially at spermatocytes and spermatids cell lineages) versus the control group. However, it was significantly decreased in medium- and high-dose aspartame-treated groups. The immunohistochemical results were confirmed by the semiquantitative RT-PCR analysis. A significant (P<0.05) increase in the mRNA level of Hsp70-2 was observed in the animals treated with low-dose aspartame. However, the mRNA levels of Hsp70-2 decreased in medium- and high-dose aspartame-treated groups (Fig .4).

## Discussion

Aspartame which is extensively used in food and medicinal products as a low-calorie sweetener, is mostly consumed by people trying to lose weights, patients with diabetes, and athletes (13). In recent decades, increased human infertility caused by toxic materials has raised concerns in human societies. In the same way, food additives and nutrition are important and influential factors in the entry of these toxic substances into the body and affect the reproductive capacity of the male sex (19). Effects of aspartame on the male reproductive parameters might be a consequence of the metabolites derived from aspartame hydrolysis during digestive and absorptive processes in the body. Studies showed that aspartame toxicity induced following oral intake is mainly related to the digestive metabolites and intestinal absorption of this substance which occurs during the metabolism of aspartame in the gastrointestinal tract by esterases and peptidases. Methanol is not metabolized in enterocytes and is rapidly introduced into the portal system of the liver and oxidized to formaldehyde by the alcohol-dehydrogenase enzyme; formaldehyde causes toxicity in most cells and tissues of the body (20). It was reported that aspartame and its metabolites potentially disturb a wide range of body processes, including amino acid metabolism, and affect the structure and metabolism of proteins, structural integration of nucleic acids and endocrine equilibria (20, 21). Many reports declared that the most destructive toxic effects of aspartame are probably related to methanol oxidation following aspartame metabolism. It was obviously indicated that receiving aspartame and subsequently the increased levels of methanol, formaldehyde and formic acid could damage the mitochondrial membrane through formation of superoxide anion and hydrogen peroxide, leading to higher levels of ROS and oxidative stress (13).

It was determined that aspartame has an effect on weight loss in humans and it can reduce weight and control obesity (22, 23). It was declared that weight loss occurs because aspartame reduces the brain's neuropeptide Y and reduction of this neuropeptide, which plays a vital role in metabolism, could reduce body weight (24). In this study, aspartame increased body weight in the highdose aspartame group, which does not match with the results of the mentioned research (22, 23). In some other studies, it was reported that aspartame inhibits an intestinal enzyme called intestinal alkaline phosphatase (IAP) which can prevent obesity, type 2 diabetes and metabolic syndrome. The results of these experiments showed that the mice that took aspartame-containing water compared to the mice without aspartame, became overweight (25).

Evidence indicates that oxidative stress can cause sperm abnormalities through various mechanisms such as inducing lipid peroxidation in sperm plasma membrane, sperm motility disorder, sperm abnormal morphology and fracture in sperm DNA (16). Also, literature shows that sperm DNA damage caused by oxidative stress increases apoptosis in immature sex cells leading to a decreases in the concentration of sperm (26). In this regard, our study showed that the use of aspartame increases sperm DNA damage by the mechanisms involved in oxidative stress induced by medium- and highdose aspartame. Previously, it was shown that using as- partame could cause an increase in the morphologically abnormal sperms that it is consistent with the results we obtained following treatment with medium- and highdose aspartame, but does not match with the effects of low-dose aspartame (27). Earlier studies showed that aspartame reduced sperm count, viability and motility in rats, which are in accordance with the findings of this study related to decreases in sperm viability and motility following administration of medium- and high-dose aspartame (13, 27).

The mechanism of action of aspartame may also be mediated via its effect on Leydig cells, which leads to a decrease in testosterone levels. With degradation and atrophy of Leydig cells under the influence of formaldehyde produced from aspartame, the levels of synthesis and secretion of testosterone decrease (28), which perfectly matched with the findings of this study that presented a significant decrease in serum testosterone level in the high-dose group of aspartame.

Besides, in order to achieve insight into the delicate in vivo oxidants/antioxidants balance, measurement of TAC could be proper. High polyunsaturated acid ratio in testes and sperm causes the male reproductive system to be susceptible to oxidative stress. The collaboration of antioxidant enzymes, SOD, CAT and GSH-Px, in cleansing ROS causes a protection of tissues and cells from oxidants’ harmful effects. So, even minor changes in normal contents of the mentioned enzymes could result in susceptibility of biomolecules to oxidative damages and so disturbances in the defense shield of the body (15). In this study, aspartame could reduce the levels of CAT, SOD and TAC in high-dose group which is supported by earlier reports (1, 11). In the defense against oxidative damages, GSH-Px has an important role by using glutathione as the reducing substrate and through catalyzing the reduction in a variety of hydroperoxides (15). We observed that administration of aspartame to mice for 90 days could dose-dependently reduce GSH-Px activity which is compatible with some earlier reports showing the ability of aspartame in reduction of GSH-Px through possessing oxidizing power (1, 11). Receiving aspartame and then increased levels of methanol and creation of formaldehyde could induce the formation of superoxide anion and hydrogen peroxide which can cause damage to the mitochondrial membrane and by inducing lipid peroxidation, could induce damage to the cell membranes (13). Increased levels of NO and MDA in the mice receiving aspartame were shown in some earlier reports (1, 11, 13), which are consistent with the results of medium- and high-dose aspartame but not the low dose, in this study.

Under different stress conditions, Hsp70-2 plays an important role in homeostasis although under physiological conditions, it is usually involved in assembling intracytoplasmic proteins. Also, biosynthesis of Hsp70-2 protein could be directly changed depending on the free radicals generation ratio in testicular tissue and depending on androgen withdrawal, it might be altered indirectly. In our study, immunohistochemical and semi-quantitative RT-PCR assessment indicated that in low-dose aspartame-exposed animals, the expression of *Hsp70-2* increased against the control group. However, medium- and high-dose aspartame-treated animals revealed significantly reduced expression of Hsp70-2 both at immunohistochemical and mRNA levels. To better understand the molecular changes at Hsp70-2 level, one should note that Hsp70-2 protein is a stress responder, and based on the intensity of the stress, it exerts homeostatic role. Therefore, in case of increasing stressors, based on its protein nature, Hsp70-2 can be peroxidated. Taking together, minding the increased Hsp70-2 expression in low-dose group, we can suggest that it exerted a homoeostatic characteristic, and based on its reduction in medium- and high-dose aspartame-administered groups, it can be concluded that due to increasing impact of stressors, pre-existing and newly synthetized Hsp70-2 proteins were peroxidated and the immunohistochemical technique failed to detect the protein. Concerning mRNA content, it is well-established that free radicals degenerate the DNA and mRNA backbones. Therefore, it is possible to suggest that due to the increasing amount of stressors, the DNA and mRNA contents of the cell were attacked and through this mechanism, the RT-PCR analysis showed diminished Hsp70-2 mRNA (29). Based on the obtained results, it could be deduced that with low-dose aspartame-induced NOS/ROS stress and androgen depletion or lower stress, in order to control the stress-induced derangements in testicular tissue, the over expression of Hsp70-2 happened. Nevertheless, the mechanisms of aspartame action at higher doses, were different. In fact, Hsp70-2 and different stimulant agents, such as NO, free radicals and superoxide affect the cellular protein structures adversely (30). Also, it could be concluded that significant reductions in total RNA and protein levels besides decreasing biosynthesis and mRNA levels of Hsp70-2, could prove that in the animals receiving high-dose aspartame, its aloneinduced damage in association with ROS/NOS-induced impairments could result in such damages to Hsp70-2 at the protein and RNA levels. In a study, it was shown that lacking Hsp70-2 in spermatocytes, caused interruption in their meiosis and they were deleted by apoptosis subsequently (29). So, it might be suggested that severe damages which were observed at spermatocyte cell levels (marked with diminished Johnsen’s criteria), induced by the aspartame were induced through affecting the expression and/or biosynthesis of Hsp70-2. Besides, expression of Hsp70-2 and its function are altered during late stages of spermiogenesis process and it could be associated with spermatid-specific-DNA-packing proteins. In fact, synthesis of protamines 1 and 2 and DNA-packing transition proteins 1 and 2, often depends on *Hsp70-2* chaperones expression (29, 31). They could provide cytoprotection against a great number of stressors and stress hormones, including corticosterone and protect cells from stress or harmful conditions (32).

While Hsps are considered regulators of apoptosis, because of this fact that the oxygen radical induced synthesis of stress proteins could result in oxidative stress tolerance, it seems that Hsp plays a role in protecting of the oxyradical-induced changes (33). Based on the obtained results, it might be concluded that the reduction induced by aspartame during spermatogenesis could be due to induction of apoptosis in spermatogenic germ cells. These results confirm apoptotic effects of aspartame, which were reported in earlier studies (7, 12, 29).

In several studies, assessment of histomorphometric parameters of testicular tissue is considered an appropriate approach for evaluating the extent of damage to this organ (15, 16). Aspartame and its metabolites such as formaldehyde, appear to change the histomorphometric parameters of testicular tissue through inducing oxidative stresses (13, 34). In this study, aspartame caused a decrease in histomorphometric parameters of testicular tissue in medium- and high-dose aspartame. In this regard, and in confirmation of the findings of this study, recent investigations also showed that aspartame and formaldehyde could induce a reduction in the Johnsen’s criteria, the diameter of the seminiferous tubules, the height of germinal epithelium and the number of Leydig and Sertoli cells (15, 35, 36).

The alkaline phosphatase enzyme plays an important role in cellular processes. Cell membrane damage results in the release of this enzyme in the cell and ultimately, in the serum. Thus, alkaline phosphatase enzyme measurement is used as an indicator for testicular tissue changes (37). Consistent with some earlier reports, in this study, dose dependent aspartame intake could increase the amount of alkaline phosphatase enzyme in testis tissue sections. Under healthy conditions, spermatogenesis series cells on the basal lamina of seminiferous tubules, possess carbohydrate sources, while the cells near the luminal space of the seminiferous tubules use lipids for their metabolism. In cases where the metabolic cycle is impaired, subsequently, cell metabolism also changes. In these circumstances, the cells use other food sources in the environment for metabolism. The results of this study showed that in testicular tissue of the mice receiving aspartame, PAS reaction (carbohydrate particles) decreases in Leydig cells and spermatogenesis series cells. These results indicate an imbalance in the metabolism of testicular tissue cells under the influence of aspartame which is consistent with other investigations in this field. In Sudan black B staining, in the present study, plenty of dense and dark granules were observed in the cytoplasm of Leydig cells, Sertoli cells and spermatogenesis series cells especially in medium- and high-dose aspartame groups. Presence of dark brown granules in the cytoplasm of Leydig and Sertoli cell adjacent to the basement membrane of the atrophied seminiferous tubules, were more obvious in Sudan black B staining which is compatible with some other studies (37, 38). Collagen fibers are studied by Masson's trichrome staining in various tissues; this study also showed that in the control group, testicular capsule had the lowest density of collagen fibers and the lamina propria in the vicinity of seminiferous tubules, showed some bundles of collagen fibers as a blue layer. The amount of these collagen strands in lamina properia of seminiferous tubules did not show any obvious changes in aspartamereceived groups, compared to the control group. Nevertheless, earlier studies indicated that formaldehyde increased the amount of collagen fibers in rats testicular tissue (34, 39). The effects of aspartame consumption result in excessive free radicals (ROS/RNS) production through different ways. The sperm abnormalities occurring due to induction of oxidative stress could affect different features of the involved cells. Consumption of aspartame affects the mitochondrial membrane integrity and leads to oxidative stress. Also, aspartame could induce some cellular disorders such as a reduction in their distribution as well as decrease in Hsp70-2 expression, damage to the cellular protein, severe damage to DNA and homeostasis contents including chaperones that in turn leads to severe oxidative stress. Aspartame, affects the Leydig cells, which induces a considerable decrease in testosterone level, and consequent dysfunction of Sertoli cells through impairing their physiological activities leading to oxidative stress, by increasing cellular apoptosis. Finally, all of the mentioned pathways will result in; increasing damage to sperm DNA, reducing sperm motility and viability and also impairing chromatin condensation (Fig .S2, See Supplementary Online Information at www.ijfs.ir).

## Conclusion

The findings of this study suggest that aspartame due to increased production of free radicals, induction of oxidative stresses and weakening the antioxidant defense system, could induce some disorders related to histomorphometric and serum parameters, increasing oxidative and nitrosative stress and down-regulating chaperone Hsp70-2 expression/biosynthesis, sperm quality and histochemical changes in medium- and highdose groups of mice. However, the results of the lowdose aspartame did not significantly differ from the control group's results and did not show any damages observed in the two other groups. Nonetheless, confirmation of the toxicity of aspartame in male reproductive system requires more extensive experimental studies, as well as clinical trials.

## Supplementary PDF


