# Supplementary material for: Long-Term Effect of Aspartame on Male Reproductive System: Evidence for Testicular Histomorphometrics, Hsp70-2 Protein Expression and Biochemical Status
Source: Int J Fertil Steril. 2020 Jul 15;14(2):91–101. doi: 10.22074/ijfs.2020.6065 (PMC7382684; doi:10.22074/ijfs.2020.6065)
Supplement: Supplementary file 1 [file Int-J-Fertil-Steril-14-91-s01.pdf]

Int J Fertil Steril
International Journal of Fertility & Sterility
ISSN:2008-076X eISSN:2008-0778

Supplementary Information for

Long-Term Effect of Aspartame on Male Reproductive System: Evidence for Testicular Histomorphometrics, Hsp70-2 Protein Expression and Biochemical Status

Hojat Anbara, Ph.D.[1], Mohammad Taghi Sheibani, Ph.D.[1*], Mazdak Razi, Ph.D.[2]

1. Department of Basic Sciences, Faculty of Veterinary Medicine, University of Tehran, Tehran, Iran
2. Department of Comparative Histology and Embryology, Faculty of Veterinary Medicine, Urmia University, Urmia, Iran

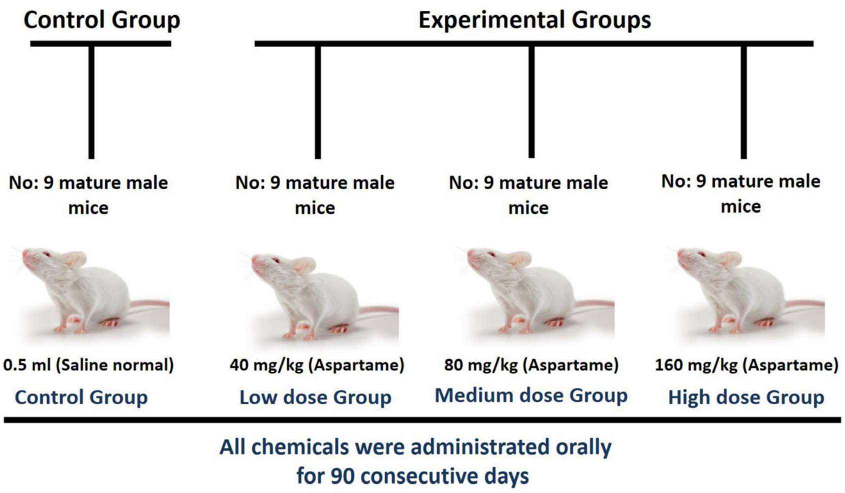

Fig.S1: Schematic view of grouping of the animals and treatment time.

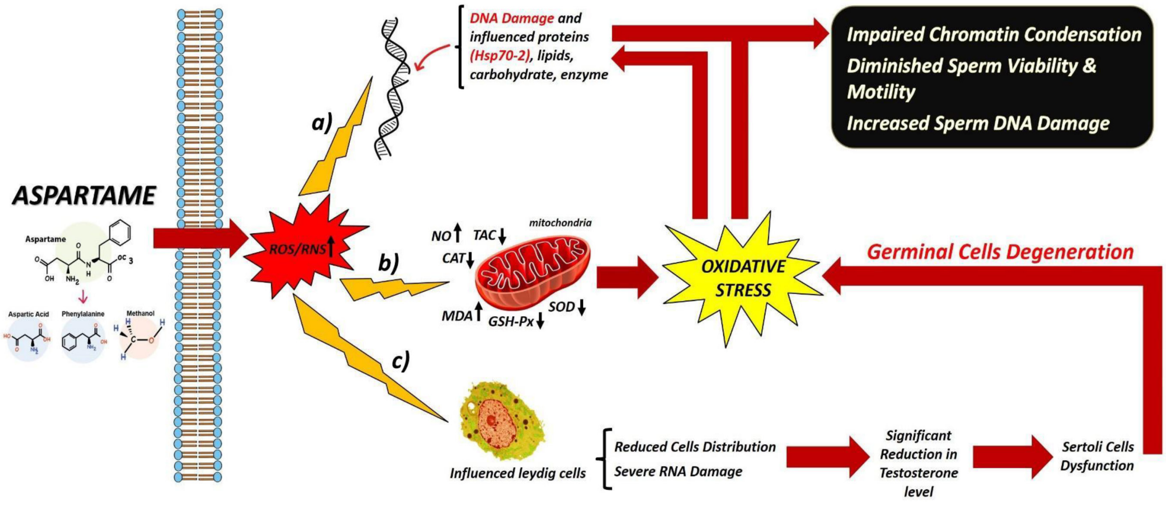

Fig.S2: Aspartame intake, by producing excessive free radicals (ROS/RNS), affect sperm characteristics in three different pathways inducing oxidative stress; a) direct effects on the cellular proteins, lipids, carbohydrates, and enzymes and inducing DNA damage, b) affecting mitochondrial membrane integrity and consequent oxidative stress and, c) it affects Leydig cells, resulting in a significant decrease in testosterone level, dysfunction of Sertoli cells and consequent oxidative stress which affect cellular ingredients. All these pathways result in impaired chromatin condensation, reduced sperm motility and viability and increased damage to sperm DNA. ROS; Reactive oxygen species and RNS; Reactive nitrogen species.

Received: 9/August/2019, Accept: 28/December/2019
***Corresponding Address: P.O.Box: 141556453, Department of Basic Sciences, Faculty of Veterinary Medicine, University of Tehran, Tehran, Iran**
Email: sheybani@ut.ac.ir
